# Identification of G protein-coupled receptor signaling pathway proteins in marine diatoms using comparative genomics

**DOI:** 10.1186/1471-2164-14-503

**Published:** 2013-07-24

**Authors:** Jesse A Port, Micaela S Parker, Robin B Kodner, James C Wallace, E Virginia Armbrust, Elaine M Faustman

**Affiliations:** 1 Department of Environmental and Occupational Health Sciences, School of Public Health, University of Washington, Seattle, WA, USA; 2 Center for Environmental Genomics, School of Oceanography, University of Washington, Seattle, WA, USA

**Keywords:** Cell signaling, Diatom, Environment, G protein-coupled receptor, Human health, Ocean

## Abstract

**Background:**

The G protein-coupled receptor (GPCR) signaling pathway plays an essential role in signal transmission and response to external stimuli in mammalian cells. Protein components of this pathway have been characterized in plants and simpler eukaryotes such as yeast, but their presence and role in unicellular photosynthetic eukaryotes have not been determined. We use a comparative genomics approach using whole genome sequences and gene expression libraries of four diatoms (*Pseudo-nitzschia multiseries*, *Thalassiosira pseudonana*, *Phaeodactylum tricornutum* and *Fragilariopsis cylindrus*) to search for evidence of GPCR signaling pathway proteins that share sequence conservation to known GPCR pathway proteins.

**Results:**

The majority of the core components of GPCR signaling were well conserved in all four diatoms, with protein sequence similarity to GPCRs, human G protein α- and β-subunits and downstream effectors. There was evidence for the Gγ-subunit and thus a full heterotrimeric G protein only in *T. pseudonana*. Phylogenetic analysis of putative diatom GPCRs indicated similarity but deep divergence to the class C GPCRs, with branches basal to the GABA_B_ receptor subfamily. The extracellular and intracellular regions of these putative diatom GPCR sequences exhibited large variation in sequence length, and seven of these sequences contained the necessary ligand binding domain for class C GPCR activation. Transcriptional data indicated that a number of the putative GPCR sequences are expressed in diatoms under various stress conditions in culture, and that many of the GPCR-activated signaling proteins, including the G protein, are also expressed.

**Conclusions:**

The presence of sequences in all four diatoms that code for the proteins required for a functional mammalian GPCR pathway highlights the highly conserved nature of this pathway and suggests a complex signaling machinery related to environmental perception and response in these unicellular organisms. The lack of evidence for some GPCR pathway proteins in one or more of the diatoms, such as the Gγ-subunit, may be due to differences in genome completeness and genome coverage for the four diatoms. The high divergence of putative diatom GPCR sequences to known class C GPCRs suggests these sequences may represent another, potentially ancestral, subfamily of class C GPCRs.

## Background

The G protein-coupled receptor (GPCR) superfamily represents one of the largest and most diverse families of proteins in mammals and is found in nearly all multicellular life [1,2]. These proteins are cell-surface receptors that play a major role in signal transduction and perception of and response to the environment. GPCRs are divided into five highly diverged families: *Rhodopsin*/class A, *Secretin*/class B, *Adhesion*/class B, *Glutamate*/class C and *Frizzled/Taste2*/class F [3]. GPCR sequences within these families can share less than 25% identity between species [4]. GPCRs bind a diverse array of ligands including proteins, lipids, neurotransmitters, calcium, odorants, and other small molecules [1]. In vertebrates, GPCR signaling networks are associated with neurotransmission, cellular metabolism, secretion, cellular differentiation and growth, inflammatory and immune responses, smell, taste and vision [5]. All GPCRs share a core seven transmembrane α-helical region with an extracellular ligand binding domain that is coupled intracellularly to a G protein heterotrimer composed of α, β and γ subunits. GPCR activation leads to the exchange of GDP for GTP by a G protein, and G protein subunits then interact and regulate effector molecules (e.g. calcium, adenylyl cyclase, phospholipase C, phosphodiesterases, protein kinases), activating further downstream signaling pathways such as the mitogen-activated protein kinase (MAPK), phosphoinositide-3 kinase (PI3K)-Akt and NF-kappaB pathways that ultimately activate transcription factors that affect gene expression and regulation [6,7]. Many of these scaffolding and signaling proteins mediate signal transduction in other intracellular pathways in eukaryotes and thus are highly conserved. The importance of these receptors is exemplified by the fact that 3-4% of human genes code for GPCRs and that nearly 30% of all currently marketed drugs target these receptors [8]. Numerous endocrine and sensory-related diseases are associated with GPCR mutations in humans [9].

Despite the crucial importance of GPCR signaling in metazoa, the prevalence and function of these proteins in non-model organisms such as unicellular photosynthetic eukaryotes is not well understood. Diatoms are a major class of eukaryotic phytoplankton found throughout the world’s oceans that play a crucial role in primary production and nutrient cycling and serve as a base for marine food webs [10]. Diatoms are also responsible for forming large phytoplankton blooms that in some cases can be toxic to humans, marine mammals and seabirds [11,12]. While the molecular mechanisms by which diatoms perceive and respond to their surrounding environment have not been resolved, previous findings suggest a role for cell surface receptors linked to intracellular signaling pathways. For example, exposure to osmotic, shear or nutrient (iron) stress in culture leads to changes in cytosolic Ca^2+^ concentrations in the diatom *Phaeodactylum tricornutum*[13]. The presence of a chemical-based defense system in *P. tricornutum* and *Thalassiosira weissflogii* has also been reported in which these diatoms respond to challenge via diatom-derived aldehydes triggering Ca^2+^ and nitric oxide release [14]. This “stress surveillance system” may function in cell-cell communication across diatom populations to detect damaged or stressed cells resulting from phytoplankton competitors and other ecological or physical stressors. These findings are important when considering environmental perception and response as alterations in Ca^2+^ homeostasis are a hallmark of signal transduction activation throughout the eukaryotes [15]. Levels of the second messenger cAMP have also been shown to change in cultures of *P. tricornutum* following exposure to elevated carbon dioxide levels [16]. While there is sequence evidence for putative GPCR signaling pathway proteins in the *Thalassiosira pseudonana*[17-19] and *P. tricornutum*[20] genomes, the role GPCR signaling may play in regulating environmental perception and response in diatoms warrants more detailed investigation.

Here we use an *in silico* approach to probe the genomes of *Pseudo-nitzschia multiseries* [http://genome.jgi-psf.org/Psemu1/Psemu1.home.html], *T. pseudonana*[17], *P. tricornutum*[20] and *Fragilariopsis cylindrus* [http://genome.jgi-psf.org/Fracy1/Fracy1.home.html] for translated nucleotide sequences with similarity to known GPCR signaling pathway proteins. We also probe expressed sequence tag (EST) libraries for each diatom to determine whether these genomic sequences are actively expressed in laboratory isolates. Our rationale for emphasizing sequence comparisons between diatoms and higher eukaryotes is three-fold. First, the GPCR signaling pathway is well-characterized in mammals compared to less well-studied, non-model organisms and thus the functions of putative homologs are better understood in this system. While model organisms such as yeast provide valuable insight into potential GPCR signaling mechanisms in mammalians, yeast and humans are found in the same eukaryotic supergroup, and thus other unicellular systems with different evolutionary histories found outside this supergroup would allow for further comparative analyses of GPCR signaling pathway diversity. Secondly, diatoms must rapidly sense and respond to multiple environmental changes, many of which are likely mediated by receptor-based signaling pathways. As major contributors to ocean productivity and carbon cycling, diatoms may play a critical role in the changing ecosystems of the future ocean, and thus understanding the breadth of their ability to sense and respond to environmental changes may be crucial to predicting their future success. Lastly, from a human health perspective, a better understanding of GPCR conservation and functionality in other organisms may provide further insight into the importance of these receptors as extracellular or environmental sensors and as pharmacological and human disease relevant targets.

The goal of this study is thus to provide a comprehensive analysis of the GPCR signaling repertoire and its potential functionality in sequenced diatoms by using a suite of bioinformatic tools aimed at annotating the genomes of non-model organisms. We hypothesize that the conservation of this pathway in diatoms may reflect shared mechanisms of environmental response related to GPCR signaling across the eukaryotes.

## Results

### GPCR signaling pathway analysis

**Figure 1:**
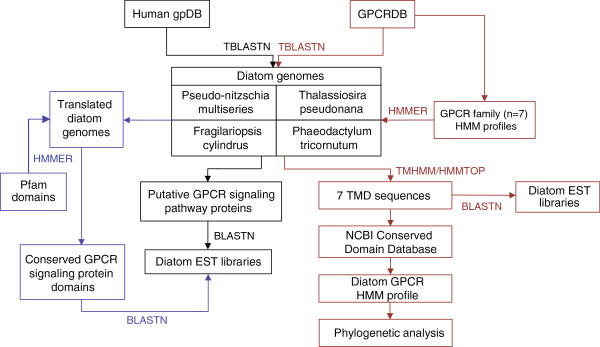
**Data analysis framework for investigating the G protein-coupled receptor (GPCR) signaling pathway in diatoms.** The diatom genomes were first TBLASTN searched against the human gpDB database to identify potential GPCR signaling pathway proteins (black). Selected conserved protein domains for those GPCR signaling pathway proteins that had no BLAST similarity to the diatoms were extracted from the Pfam v.26.0 database and HMMER searched against the translated diatom genomes (purple). Diatom genomic sequences with matches to the human gpDB or GPCR signaling protein Pfam were then searched against the diatom EST libraries. The GPCRDB was TBLASTN searched against the diatom genomes to identify putative diatom GPCRs (red). GPCRs were also identified by downloading sequence alignments for the GPCR families from the GPCRDB (classes A, B and C) to use as seed alignments for HMM searches against a custom microeukaryote database (red). Identified GPCRs were then further characterized using transmembrane domain (TMD) region and conserved domain analyses. Diatom GPCRs were also searched against the respective diatom EST libraries. A seed alignment was generated using the TMD regions of the putative diatom GPCRs and converted to an HMM profile to recruit related sequences from the custom microeukaryote database and GenBank. Phylogenetic analysis was then performed. BLAST, basic local alignment search tool; EST, expressed sequence tag; GPCRDB, G protein-coupled receptor database; HMM, hidden markov model; HMMTOP, Hidden Markov Model for Topology Prediction; TBLASTN, protein query versus translated nucleotide BLAST; TMHMM, transmembrane hidden markov model.

**Figure 2:**
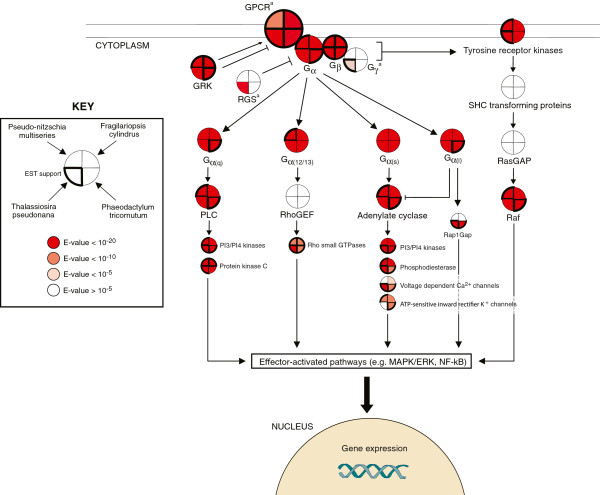
**Human G protein-coupled receptor (GPCR) signaling pathway proteins that are conserved across the diatom genomes and EST libraries.** Conservation is based on BLAST sequence similarity criteria consisting of an e-value <10^-5^, <10^-10^, or 10^-20^ over a minimum alignment length of 100 amino acids unless specified otherwise. Homology to the G protein γ-subunit and RGS was identified using the Pfam database and HMMER. Note that this is an abbreviated representation of the GPCR signaling pathway that includes the primary components and a selection of the major downstream effectors. ^a^Due to the absence of human homologs, the BLAST search space for GPCRs, the Gγ-subunit and RGS was expanded to include all organisms. Gα(i), Gi alpha subunit protein; Gα(q), Gq alpha subunit protein; Gα(s), Gs alpha subunit protein; Gα(12/13), Gi alpha subunit protein 12/13; GRK, G protein-coupled receptor kinase; MAPK/ERK, mitogen-activated protein kinases/extracellular signal-regulated kinases; NF-kB, nuclear factor-kappa B; PI3/PI4 kinases, phosphatidylinositide 3- and 4- kinases; PLCβ, phospholipase C beta; Raf, RAF proto-oncogene serine/threonine-protein kinase; Rap1GAP, Rap1-GTPase activating protein; RasGAP, Ras GTPase activating protein; RhoGEF, Rho guanine nucleotide exchange factor; RGS, regulator of G protein signaling.

Using the analysis framework shown in Figure 1, we first searched the diatom genomes for evidence of GPCR signaling pathway proteins, many of which are expected to be highly conserved across eukaryotes (Table 1). Genomic matches were then searched against the respective diatom EST library. Based on sequence similarity to human proteins from the human gpDB database [21], all four diatoms have genes coding for core components of the GPCR signaling pathway. These putative proteins share approximately 25-50% sequence identity to human GPCR pathway proteins when considering alignment lengths >100 aa (Figure 2 and Additional file 1: Table S1).

**Table 1:** **Known functions of the major proteins involved in the mammalian G protein-coupled receptor (GPCR) signaling pathway as defined by the human gpDB**[21]

**Protein category**	**Function**
Adenylate cylcase	Transmembrane protein regulated by G protein; catalyzes formation of the second messenger cyclic adenosine monophosphate (cAMP) from ATP.
ATP-sensitive inward rectifier K + channels	Regulated by G proteins and GRKs. Activation leads to hyperpolarization and reduction of membrane excitability.
G protein (Gα,β,γ)	Heterotrimeric protein composed of α,β, and γ subunits; activated by GPCR to bind to and activate/deactivate various effectors (e.g. second messengers); amplifies receptor signal. The α-subunit is divided into several sub-types that perform different functions by activating various effector proteins: α(q) activates PLC, α(s) activates the cAMP-dependent pathway via activation of AC, α(i) inhibits AC and thus cAMP production and α(12/13) activates Rho GTPases.
G protein-coupled receptor (GPCR)	Cell surface receptor; binds agonist/ligand, catalyzing exchange of GDP for GTP on G protein; dissociates and activates G protein subunits.
GPCR kinase (GRK)	Regulates GPCR activity via phosphorylation; desensitizes the receptor signal.
Phosphodiesterase	Degrades the phosphodiester bond in the second messengers cAMP and cGMP; terminates receptor signal.
Phosphoinositide-3 kinase (PI3K)	Recruited to the cell membrane following GPCR activation; binds G protein and initiates assembly of signaling complexes and priming of protein kinase cascades; hyperactivation of this pathway has been associated with cancer and diabetes.
Phospholipase C	Catalyzes hydrolysis of phospholipids to generate the second messengers inositol 1,4,5-triphosphate (IP3) and diacylglycerol (DAG); amplifies signal by stimulating Ca^2+^ release and protein kinase activation.
Protein kinase C	Regulates signal transduction; activated by G proteins or increases in cytosolic Ca^2+^; phosphorylates a wide variety of proteins including small GTPases and MAPKs
Raf	Member of the serine/threonine-specific protein kinases that functions downstream of the Ras subfamily. Raf activates the MAPK/ERK pathway.
Rap1GAP	Encodes a GTPase-activating protein that down-regulates the activity of the RAP1 protein. RAP1 is a Ras subfamily protein.
RasGAP	Stimulates the GTPase activity of Ras, thereby inactivating Ras. Ras acts as a molecular switch, functioning within a signal transducing cascade of reactions.
Regulator of G protein signaling (RGS)	Inactivates G protein, leading to rapid turnoff of GPCR signaling. RGS promotes GTP hydrolysis by the G protein α-subunit.
RhoGEF	Structural domain of guanine nucleotide exchange factors for Rho-like GTPases that controls Rho signaling by mediating GDP release from Rho and replacing with GTP.
Rho small GTPases	Family of small signaling G proteins that are homologous to Gα subunit but are monomeric in structure. These proteins interact with and activate effector proteins that mediate downstream signaling.
SHC transforming proteins	Src homology 2 domain containing protein. Links activated tyrosine receptor kinases to the Ras pathway.
Tyrosine receptor kinases	Cell surface receptor that link GPCRs to the Ras-MAPK pathway. Activated by the G protein βγ subunit and in turn stimulates the Ras subfamily proteins.
Voltage dependent Ca2+ channels	Modulates calcium influx into the cell. GPCRs play critical role in negative feedback to inhibit the activity of these channels via direct interaction with the G protein βγ subunit.

### G protein

G protein α- and β-subunits were well-conserved across all four diatoms and were verified with EST support (Figure 2 and Additional file 1: Table S1). All four classes of the G α-subunit were conserved, including α_q_, α_12/13_, α_s_ and α_i_. The level of sequence similarity was slightly higher for diatom G protein α- and β-subunits and human homologs than for known Viridiplantae G protein subunits and human G proteins (Additional file 1: Table S1). There was no sequence similarity to the G protein γ-subunit in any of the diatoms using BLAST with human Gγ-subunits or with the canonical plant Gγ-subunits from *Arabidopsis thaliana* (GI: 12034688 and 14625852) and *Oryza sativa* (GI: 46357950 and 42409262). This may be expected given that the Gγ-subunit has been shown to be highly divergent between species and among model organisms such as *Arabidopsis*, rice, corn and soybean [22], Furthermore, the Gγ-subunit is a small protein (~70-100 aa), and thus conventional BLAST parameters may miss more distant homologs. Therefore, to increase our search sensitivity, the Pfam Gγ subunit GGL domain (PF00631) was used to screen conceptually translated diatom genome sequences. The GGL domain is found within the Gγ subunit across a wide range of species. The HMMER search identified an open reading frame (ORF) of 71 amino acids in *T. pseudonana* with similarity to the GGL domain (E = 10^-8^) (Figure 2 and Additional file 1: Table S1). Furthermore, this ORF contained the highly conserved canonical C-terminal CaaX box involved in post-translational processing of the Gγ-subunit [22]. The corresponding genomic region on chromosome 2 in *T. pseudonana* (bp 1358730–1358942) had >99% similarity to four ESTs. The 71 aa ORF had the best match to a hypothetical protein in another stramenophile (*Albugo laibachii*) that also contains the GGL domain and CaaX box. Other matches containing the GGL domain fell into either stramenophile or amoebozoa clades and had E-values as high as 9.7, demonstrating the need for alternative approaches to standard BLAST criteria.

### RGS

Because regulators of G protein signaling (RGS) have been shown to have high affinity for the Gβ-subunit and therefore may substitute for the Gγ-subunit in the Gβγ complex [23], we searched the translated diatom genomes for the RGS protein. We compiled a small database of 100 human RGS variants to search against the diatom genomes using TBLASTN. The only significant match was to *T. pseudonana* with an E-value of 10^-12^. To further verify the absence of RGS in the other three diatoms, the conserved ~125 aa RGS core domain (PF00615), which is present in all RGS proteins, was HMMER searched against the translated diatom genomes. *T. pseudonana* again had a significant match (E < 10^-20^) while matches to the other diatoms had E-values >10^-5^ and either partial or split RGS domains.

### Effector proteins

The Gα-subunit and Gβγ complex activate effector proteins, leading to stimulation or inhibition of second messengers such as cAMP, cGMP, diacylglycerol and inositol (1,4,5)-trisphosphate and phosphatidyl inositol (3,4,5)-triphosphate as well as opening or closing ion channels and regulation of intracellular Ca^2+^. The four different classes of the Gα-subunit interact with their respective downstream effectors including phospholipase C (PLC), Rho family GTPase signaling proteins and adenylate cyclase. Except for RhoGEF, these effector enzymes were present in all four diatoms based on sequence similarity. EST support was also available for PLC and adenylate cyclase in *P. multiseries* and *P. tricornutum* and for the Rho small GTPases in all four diatoms. The presence of adenylate cyclase, phosphodiesterase and protein kinase C suggest a role for cAMP signaling in the diatoms, and similarly PLC, protein kinase C and Ca^2+^ channels support a role for Ca^2+^ signaling. Other than Raf, no similarity was detected for Ras-related proteins within the tyrosine kinase pathway. Receptor tyrosine kinase was present in the diatom genomes and supported by EST evidence in *P. multiseries* and *T. pseudonana*. Tyrosine kinases have been shown to mediate GPCR-induced Ras activation. GPCR kinase, which regulates the activity of GPCRs, was also conserved (E <10^-50^) in all four diatom genomes and also in the EST libraries except for *T. pseudonana*.

### GPCRs

Due to the divergence of GPCR sequences, the BLAST search space was expanded to include all known and predicted eukaryotic GPCRs within the G protein-coupled receptor database (GPCRDB) (http://www.gpcr.org/7tm/) [24]. This approach resulted in 5 *F. cylindrus,* 5 *P. multiseries*, 4 *P. tricornutum* and 2 *T. pseudonana* candidate GPCR sequences with correct transmembrane domain (TMD) orientation and the strongest similarity to the class C GPCR superfamily (Table 2). The best protein matches for the putative GPCR sequences for *P. multiseries* and *F. cylindrus* were to a class C receptor from the unicellular amoebae *Dictyostelium discoideum* (Additional file 1: Table S1). The best matches for the putative GPCR sequences from *T. pseudonana* and *P. tricornutum* were to GABA_B_ receptors (class C family) from a mosquito and *X. tropicalis*, respectively. For reference, the percent identity of the diatom putative GPCR sequences to their best human match (22-26%) was similar to that of the known class C receptor from *D. discoideum* to its best human match (27%). *P. tricornutum* GPCR1a and GPCR1b appear to be splice variants of the same gene.

**Table 2:** Expressed sequence tag (EST) data for the putative diatom G protein-coupled receptors (GPCRs)

**Sequence**	**Accession no. (JGI/NCBI)**	**Length (aa)**	**% EST coverage (≥98% identity)**	**No. of EST sequences**	**EST coverage region (aa) (≥98% identity)**	**Culture treatment**	**Reference**
*P. multiseries*							
GPCR1	240179	906	45	1	499-906	Phosphate starvation	^1^JGI
GPCR2	117604	1003	17	1	616-819	Domoic acid-producing conditions	^2^
GPCR3	179685	521	No hits	N/A	N/A	N/A	N/A
GPCR4	288475	461	No hits	N/A	N/A	N/A	N/A
GPCR5	293132	887	No hits	N/A	N/A	N/A	N/A
*F. cylindrus*							
GPCR1	242057	911	No hits	N/A	N/A	N/A	N/A
GPCR2	241103	1020	No hits	N/A	N/A	N/A	N/A
GPCR3	236496	1102	No hits	N/A	N/A	N/A	N/A
GPCR4	266398	899	30	2	32-305	Osmotic stress, pooled RNA (5 treatments)	^3^
GPCR5	253555	257	No hits	N/A	N/A	N/A	N/A
*P. tricornutum*							
GPCR1A	219114538	688	100	64	1-688	16 different treatments	[20]
GPCR1B	219117625	626	100	64	1-619	16 different treatments	[20]
GPCR2	219114407	799	69	8	1-552	16 different treatments	[20]
GPCR3	219113755	1111	86	22	1-228/378-1111	16 different treatments	[20]
GPCR4	219113191	1012	74	18	1-125/366-986	16 different treatments	[20]
*T. pseudonana*							[25]
GPCR1	224005915	627	No hits	N/A	N/A	^5^Upregulated during silaffin-like response	[25]
GPCR2	224006404	846	26	1	570-792	Iron-limited cells	^4^JGI

*N/A,* not applicable.

^1^http://genome.jgi-psf.org/Psemu1/Psemu1.home.html.

^2^Boissonneault KR, Bates SS, Milton S, Pelletier J, Housman DE: Gene discovery and expression profiling in the toxin (domoic acid)-producing marine diatom *Pseudo-nitzschia multiseries* (Bacillariophyceae) using cDNA microarrays, unpublished.

^3^Krell A, Gloeckner G: Analysis of an osmotic stress induced cDNA library of the psychrophilic diatom *Fragilariopsis cylindrus*, unpublished.

^4^http://genome.jgi-psf.org/Thaps3/Thaps3.home.html.

^5^*T. pseudonana* GPCR1 had no corresponding EST data but the sequence was found to be upregulated during transcriptome profiling under silicon starvation conditions.

**Figure 3:**
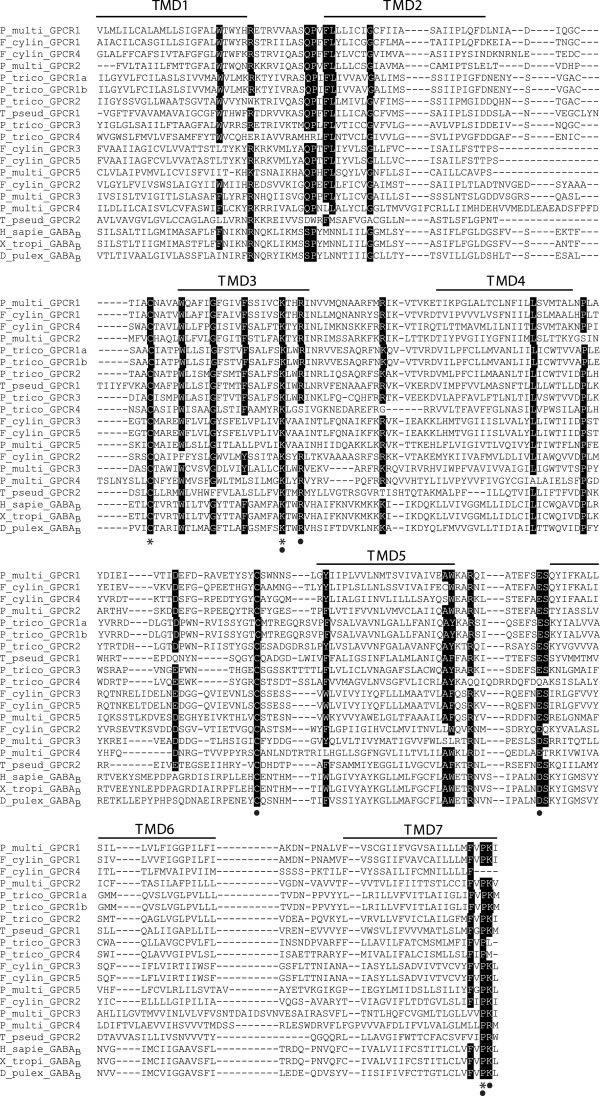
**Multiple sequence alignment for the transmembrane domain (TMD) regions of the putative diatom GPCRs.** Highlighted regions represent the most conserved residue positions among the diatom sequences. Asterisks denote residue positions conserved across all sequences and circles denote residue positions critical in family C GPCR activation or conserved across metazoan class C receptors. H_sapie_GABA_B_, Homo sapiens GABBR2 protein (GI: 34191359); X_tropi_GABA_B_, Xenopus tropicalis GABA_B_ receptor 2 (GI: 186972153); D_pulex_GABA_B_, Daphnia pulex GABA_B_ receptor (GI: 321470990).

The TMD sequences contained residues conserved across the class C GPCRs (Figure 3). The two cysteine residues in the first and second extracellular loops are postulated to be involved in disulfide bond formation [26]. The two basic residues (Lys and Arg) at the cytoplasmic face of the TMD3 and the acidic residue (Glu or Asp) at the cytoplasmic end of the TMD6 [27] have been shown to be crucial for activation of the GABA_B_ receptor subclass of class C GPCRs. The PK motif in TMD7 is also conserved in class C GPCRs [26]. Other conserved residues that may be important for receptor activation in the diatom sequences are highlighted in Figure 3.

We used a second approach to identify potential diatom GPCRs by downloading the class A, B and C GPCR alignments from the GPCRDB, and then converting these alignments to hidden markov model (HMM) profiles to search against a custom microeukaryote database (Additional file 2: Table S2) and the NCBI RefSeq database. No diatom sequences were recruited to the class A and class B alignments, while two sequences that were also identified using the BLAST approach (*F. cylindrus* GPCR5 and *T. pseudonana* GPCR2) were recruited to the class C alignment. These results further indicate that putative diatom GPCRs are likely to be highly diverged from known metazoan GPCRs and that they appear to have family C GPCR-like properties.

Based on sequence similarity searches against the diatom EST libraries, there is evidence for transcription of a number of the putative diatom GPCR sequences (Table 2). The putative *P. tricornutum* GPCR sequences had the greatest EST coverage, likely due to the larger repository of ESTs for this diatom. The majority of these ESTs detected in the four diatoms came from cultures under various stress conditions, including growth-limiting factors for *P. tricornutum*, domoic-acid producing conditions for *P. multiseries*, osmotic stress for *F. cylindrus* and iron limitation and silicon starvation for *T. pseudonana*.

**Figure 4:**
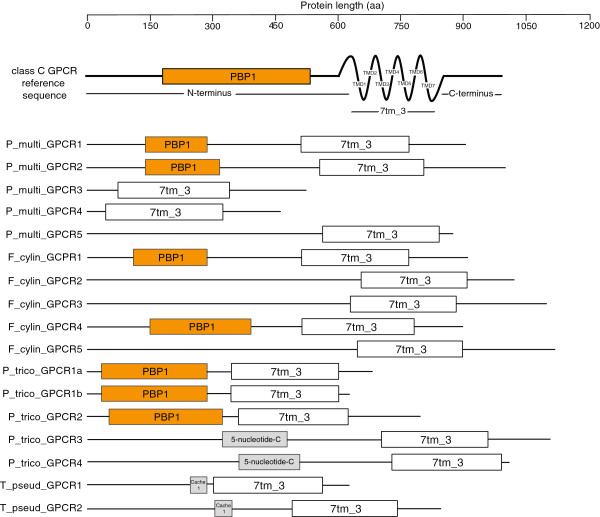
**Structure and size of putative diatom GPCRs.** A reference structure for a mammalian class C GPCR is provided for comparison. Conserved domains are boxed and include: PBP1, periplasmic binding protein domain which is considered to be the evolutionary origin of the class C GPCR ligand binding domain; 7tm_3, seven transmembrane domain of class C GPCRs; 5-nucleotide-C, associated with enzymatic degradation of sugars; Cache 1, extracellular protein domain involved in recognition of small molecules. TMD, transmembrane domain.

The length of the candidate diatom GPCR sequences ranged from 300 to over 1000 amino acids. This wide range was due to considerable size and domain variation in the N- and C-termini among the candidate class C GPCR sequences (Figure 4). Seven of these sequences contain an N-terminus domain with similarity to prokaryotic periplasmic binding proteins (PBPs), which are involved in amino acid and nutrient transport in bacteria and are considered to be the origin of the class C GPCR ligand binding domains [26]. Based on BLAST similarity, four of these seven sequences (*P. multiseries* GPCR1, *P. multiseries* GPCR2, *F. cylindrus* GPCR1 and *F. cylindrus* GPCR4) have N-termini with strongest similarity to the class C GPCRs, while the other three from *P. tricornutum* have similarity to the periplasmic component of ABC sugar transporters, which also are known to contain PBPs. We verified the completeness of the gene prediction models by searching the genomic regions upstream of the N-terminus of the diatom GPCR sequences for ORFs containing the ligand binding domain using the NCBI Conserved Domain Database (CDD). No PBP-like domains were detected in diatom GPCR sequences that were originally not predicted to contain the domain. There was also no evidence in the extracellular region upstream of the TMD for a cysteine-rich domain. This domain is found in certain class C GPCRs including metabotropic glutamate, calcium-sensing and taste receptors but absent in GABA_B_ receptors [28]. The C-termini of the putative diatom GPCRs share no similarity to other GPCRs or known proteins in general. This is not the case for other known class C GPCRs, which share sequence similarity in the C-terminus.

### Phylogeny of predicted diatom GPCRs

**Figure 5:**
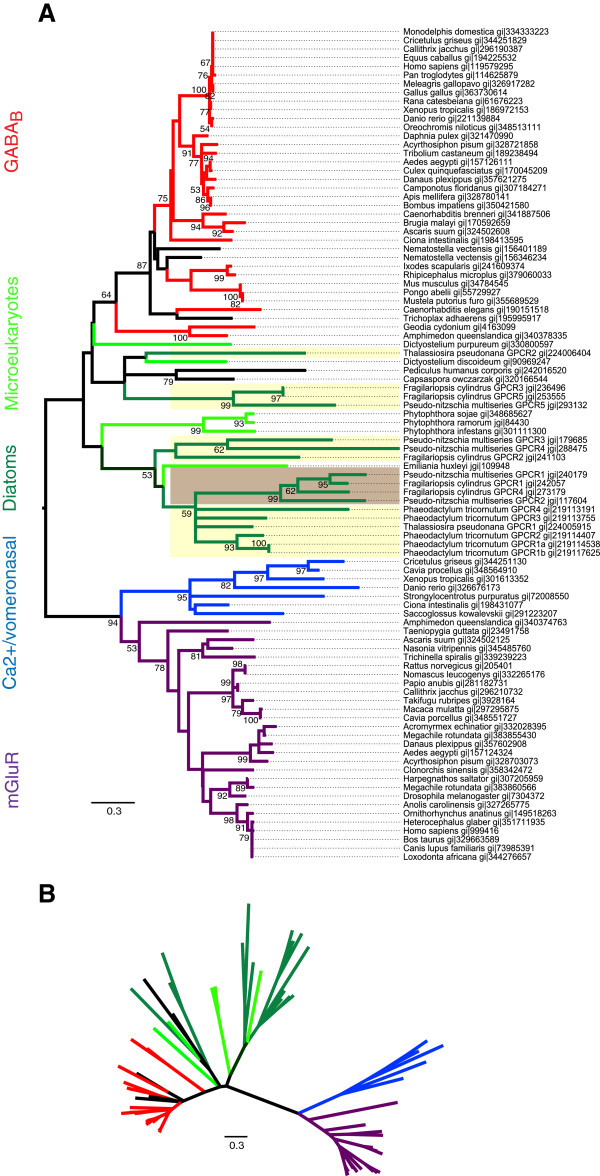
**Maximum likelihood phylogenetic trees of the putative diatom GPCRs in relation to known class C GPCRs and microeukaryotic homologs.(A)** Rooted and **(B)** unrooted trees. The unrooted tree highlights the overall genetic distance and relationships between branches. Diatom GPCR sequences identified in this study that contain the N-terminal ligand binding domain are highlighted in brown, those not containing the binding domain are highlighted in yellow. Class C GPCR subfamilies and putative diatom and microeukaryote GPCRs are color-coded: diatoms, dark green; microeukaryotes, light green; GABA_B_, red; metabotropic glutamate, purple; calcium-sensing and vomeronasal, blue; hypothetical proteins, black. Bootstrap values ≥50% were included in the tree.

To determine the phylogenetic relationship among the putative diatom GPCRs and their relationship to known GPCRs from other domains of life, the 7 TMD regions of the putative diatom GPCRs identified by BLAST were used as the seed alignment to HMM search for other GPCRs in the custom microeukaryote and NCBI RefSeq databases (Figure 1). The recruited sequences were then used to generate a tree that examined the diversity of possible diatom GPCRs among other putative microeukaryote and established GPCRs. The diatom TMD HMM profile recruited an array of class C GPCRs, with the strongest sequence similarity to the GABA_B_ receptors and to lesser extent the mGluR and calcium-sensing/vomeronasal receptors. For maximum likelihood (ML) tree construction, the wag amino acid model was found to have the highest likelihood score when comparing models with different fixed substitution rates among the different amino acid changes. We also tested a number of other amino acid models and obtained similar tree topology. Within the ML tree, the majority of diatom sequences clustered with one another and with other microeukaryotes and formed a deeply diverged sister clade to the GABA_B_ receptors albeit with weak bootstrap support (Figure 5). The seven putative diatom class C GPCR sequences that contained the PBP1 ligand binding domain grouped within a divergent clade. Within this clade, the sequences were differentiated by their N-termini, with the three *P. tricornutum* sequences that had similarity to bacterial ABC transporters grouping together and the remaining three sequences (*P. multiseries* GPCR1, *P. multiseries* GPCR2, *F. cylindrus* GPCR1 and *F. cylindrus* GPCR4) clustering together with strong bootstrap support. Other than the stramenopiles (diatoms and *Phytopthora*), microeukaryotic sequences recruited to the tree included the slime mold (*Dictyostelium sp.*) and the coccolithophore *Emiliania huxleyi*.

## Discussion

Using a comparative genomics approach, we elucidated a GPCR signaling repertoire in diatoms that points to the highly conserved nature of this signaling pathway across the eukaryotes. Specifically, sequence comparisons indicate the presence and expression of GPCRs, Gα- and β- subunits and other common downstream effectors and pathways known to be activated by GPCR signaling. There was genomic evidence and EST support for the Gγ-subunit in *T. pseudonana* only. *T. pseudonana* was also the one diatom to contain a complete RGS domain. Based on a combination of BLAST, HMM profiling and phylogenetic analyses, the putative diatom GPCRs were most similar to but still highly diverged from the class C GPCRs. Furthermore, they formed a clade basal to the GABA_B_ receptors within the class C GPCRs. There was considerable variability in the size of the putative diatom GPCRs, and seven of these sequences had N-terminus similarity to the class C ligand binding domain. A number of these putative GPCR sequences also have EST support. These putative GPCRs may potentially represent an additional group of class C sequences recovered from diatoms and other microeukaryotes that do not fit within existing class C subfamilies.

### The diatom GPCR signaling pathway repertoire

The majority of the proteins involved in the GPCR signaling pathway appear to be well conserved in the diatom genomes based on protein sequence similarity. Furthermore, gene expression data indicates that the genes coding for many of these proteins are transcribed. Despite strong conservation of the Gα- and β- subunits in all four diatoms, there was evidence for the Gγ-subunit in only *T. pseudonana*. All identified functional G proteins in other organisms are heterotrimeric, consisting of the three subunits. The β and γ-subunits function structurally as a monomer, as the two subunits cannot be dissociated [29]. The G protein βγ subunit regulates the functionality of the α-subunit in addition to mediating downstream signaling pathways. While the γ-subunits of different mammalian species can share as low as 27% sequence similarity [30], we were still not able to recover the highly conserved γ-subunit GGL domain in *P. multiseries*, *F. cylindrus* and *P. tricornutum*. Furthermore, RGS, which in some studies has been shown to act as a substitute for the Gγ-subunit [23], was also only present in *T. pseudonana*. This may simply be due to differences in genome coverage and genome size between the diatoms, as described below. Nonetheless, the structure and functionality of the G protein in diatoms requires further investigation.

The putative diatom GPCR sequences had the strongest similarity to the class C GPCRs based on TMD and in a few cases N-terminus similarity. Class C receptors are characterized by a long extracellular N-terminus (~600 aa) required for ligand binding. The diatom N-termini ranged from 40–730 aa, with the shorter domains potentially representing a truncated or absent ligand binding region and thus altered functionality. Viral GPCRs provide an example of a GPCR with a very small or absent N-terminus whose functionality is maintained through constitutive expression [31]. The N-terminus ligand binding domain required for class C GPCR activation was present in seven of the putative GPCRs. Four of these sequences (*P. multiseries* GPCR1 and GPCR2 and *F. cylindrus* GPCR1 and GPCR4) have the strongest similarity to the known class C GPCR topology, and also phylogenetically group together. Three of these candidate GPCRs (from *P. tricornutum*) had stronger similarity to the periplasmic component of bacterial ABC sugar transporters, which are in the same superfamily as the GPCR ligand binding domains. Their TMDs had typical class C structure and similarity. These sequences were also shorter than typical class C receptors (especially within the N-terminus). Taken together, these results suggest that these *P. tricornutum* sequences are class C GPCR-like but may have modified or altered binding activity. The ligand binding domain is considered to have evolved from prokaryotic periplasmic binding proteins which are involved in amino acid and nutrient transport in bacteria [26]. The lengths of the intracellular C-termini were also variable across the diatom sequences, and the C-termini shared no sequence similarity to any other proteins. This is not unexpected given the fact that the C-terminus is the least conserved region of class C GPCRs, even among orthologs [26]. The C-terminus is not required for receptor coupling to G proteins, but interactions have been shown between this region and various scaffolding proteins associated with receptor signaling, desensitization and targeting. In sum, while the TMD regions of these putative diatom GPCR sequences were conserved, further investigation into the diversity of the N- and C- termini are needed to classify these sequences. The phylogenetic analysis does indicate that there is considerable diversity even within the TMDs that distinguishes the diatom and microeukaryotic sequences from the TMDs of metazoan class C GPCRs. The fact that those diatom GPCR sequences with the ligand binding domain cluster together suggests that the TMD alone can distinguish the presence or absence of putative GPCR binding domains based on the conserved amino acid residues shown in Figure 3.

The extent and diversity of microeukaryotic sequences recovered from the HMM searches suggests GPCR signal transduction is an ancient signaling cascade retained in diverse forms of life. The putative diatom and microeukaryotic GPCRs were distantly related to the metazoan class C GPCRs and formed a series of branches basal to the metazoan GABA_B_ receptor sequences. A distant relationship between these groups of putative GPCRs is expected considering the deep evolutionary relationships of these organisms and the unlikely functional homology of the putative diatom and microeukaryote sequences to metazoan receptors. The class C GPCR subfamily profile recovered sequences from the stramenopiles (diatoms and *Phytopthora*), a haptophyte (*Emiliania huxleyi*) and slime mold (*Dictyostelium* sp.). Slime molds are evolutionarily basal to the opisthokonts, which include metazoa, and thus the presence of metazoan homologs in *Dictyostelium* is not surprising. Diatoms and haptophytes were the only photosynthetic organisms recruited by these searches. Both of these groups are products of secondary endosymbiotic events [32] and therefore derive from a more recent heterotrophic host cell than green or red algae. An example of the acquisition of a novel trait due to this secondary endosymbiotic event is the presence of the urea cycle in diatoms [33]. The urea cycle is typically associated with metazoan metabolism, and is absent in green algae and plants. The class C GPCR-like proteins in the diatoms may also reflect their evolutionary history and represent a more ancestral version of this protein family. The other photosynthetic chlorophyll a + c groups that were included in the custom database, such as dinoflagellates and cryptophytes, were not recruited to the class C subfamily profile with a HMMER e-value <10^-5^. The diatom sequences profile also did not recruit any sequences at an e-value <10^-5^ from *Aureococcus,* another microalgal stramenopile. A putative GPCR sequence was recovered from an additional slime mold but no sequence matches were recovered from the many other heterotrophic microeukaryotes for which ESTs are available or from *Naeglaria gruberii*, one of the only free living excavate-lineage genomes available. The apparent sporadic recovery of GPCR-like sequences from microeukaryotes may simply be an issue of low reference sequence availability, possibly compounded by low expression of the putative class C receptor homologs in microeukaryotes, limiting representation in EST libraries. Alternatively, the basal nature of the putative diatom and microeukaryotic class C GPCR sequences may indicate that these sequences represent a separate, possibly ancestral, family of class C GPCRs. It is possible that diatom GPCRs underwent an independent evolution after a recombination event between an ancestral class C receptor and a GPCR-like locus, or evolved directly from an ancestral class C receptor as has been proposed for plant GPCRs [34].

### Functionality of GPCR signaling in diatoms

Class C GPCRs are responsible for a vast array of physiological processes ranging from the modulation of synaptic transmission to the perception of sensory stimuli in the nervous system [35]. The primary ligands for class C receptors include the neurotransmitters glutamate and GABA. Sequences cloned from the sponge [36] and the amoeba *Dictyostelium discoideum*[37] represent the most basal class C receptors that have been identified to date. A metabotropic glutamate receptor identified in *D. discoideum* (DdmGluPR) is diverged from other characterized metabotropic glutamate receptors in multicellular organisms (Figure 5), as is also the case for the putative diatom GPCR sequences. Functionally, DdmGluPR is involved in early development of *D. discoideum*[37]. GABA produced by *D. discoideum* also functions as an intracellular signaling molecule by regulating differentiation during development through a GABA_B_ receptor homologue [38,39]. *D. discoideum* thus provides an example whereby class C receptors play an important functional role in the absence of neuronal synapses, and may therefore shed light on the functionality of potential diatom GPCRs.

While GABA has been directly detected in cultures of *P. tricornutum*[40], there has been no documented role for GABA in diatoms or algae in general. In the mammalian central nervous system, GABA is the most widely distributed inhibitory neurotransmitter [41]. It has also been found in nearly every plant and plant part examined [42]. GABA levels in plants increase several-fold in response to biotic and abiotic stresses such as heat shock, cold shock, mechanical stimulation, hypoxia, phytohormones, and water stress [43,44]. There is also support for a role for GABA in plants in contributing to C:N balance, regulation of cytosolic pH, protection against oxidative stress, self-defense, osmoregulation and cell signaling [44]. A GPCR system in diatoms involving GABA or other extracellular or intracellular ligands may function similarly in protecting the cells against abiotic and biotic stressors such as temperature or salinity changes. The coupling of GABA_B_ receptors with Ca^2+^ ion channels or Ca^2+^-sensing receptors in other organisms [45,46] suggests the potential for a GPCR to mediate the documented increase in intracellular Ca^2+^ following exposure to physical and chemical stressors in diatoms [13,14,16].

### Database limitations

Diatom genome size, coverage and completeness varied greatly between the diatoms analyzed in this study. This may have impacted our ability to profile completely the repertoire of GPCR signaling pathway proteins (e.g. Gγ-subunit, RGS) in diatoms with less sequence data available. *P. multiseries* has an estimated eight-fold larger genome size (218.7 Mbp) and *F. cylindrus* a three-fold larger genome size (80.5 Mbp) than the smallest diatom *P. tricornutum* (26.1 Mbp). The smaller diatom genomes though have greater sequence coverage (9.6× and 12.8× for *P. tricornutum* and *T. pseudonana* respectively) than the two larger diatoms (~7-8×). The genome sequences for *P. tricornutum* and *T. pseudonana* are also assigned to finished chromosomes rather than genome scaffolds. As genome and EST coverages increase and additional diatom genomes are sequenced, evidence for the GPCR signaling pathway in these organisms will likely become more complete.

## Conclusions

In summary, using a cross-species comparative genomics approach this study has found conservation at the amino acid level of many of the core proteins involved in the mammalian GPCR signaling pathway in diatoms. Sequence similarity and expression data support the presence of GPCRs, Gα- and β- subunits and other common downstream effector proteins. While evidence for the Gγ-subunit and RGS was only detected in *T. pseudonana*, the lack of these proteins in the other diatoms suggests a need for increased genome coverage or further investigation into the heterotrimeric nature of the G protein in diatoms. A number of the putative diatom GPCR sequences have transcriptional evidence under various stress conditions in culture. Phylogenetic analyses revealed the putative diatom GPCR sequences to be most similar to but deeply diverged from the class C GPCRs, and within this family they appear to form a unique clade basal to the GABA_B_ receptors. These putative diatom GPCR sequences exhibit high diversity in the N- and C- termini and have a conserved TMD region that is unique from that of metazoan class C receptors. The presence of GPCRs and GPCR signaling proteins in diatoms suggests a secondary signaling mechanism that warrants further experimental investigation to better define the functional roles of these proteins in diatoms. The confirmation of GPCR functionality in diatoms would indicate that these organisms are able to perceive and respond to their surrounding environment in a more complex manner.

## Methods

The complete genomes and filtered models for the diatoms *T. pseudonana* v3.0, *P. tricornutum* v2.0, *P. multiseries* v1.0 and *F. cylindrus* v1.0 were obtained from the DOE Joint Genome Institute (JGI) database [http://www.jgi.doe.gov/genome-projects/]. Expressed sequence tags (ESTs) for each diatom were downloaded from GenBank and supplemented with the diatom ESTs from JGI. In total we searched 77,631, 146,023, 78,091 and 24,724 ESTs for *T. pseudonana*, *P. tricornutum, P. multiseries* and *F. cylindrus* respectively.

### GPCR signaling pathway analysis

Using the Basic Local Alignment Search Tool (BLAST) program [47], the human gpDB was TBLASTN (default settings) queried against the conceptually translated genomes for each diatom to generate protein sets for *P. multiseries*, *F. cylindrus*, *T. pseudonana* or *P. tricornutum* with similarity to human GPCR signaling pathway proteins (Figure 1). The human gpDB is a curated collection of human GPCRs, G proteins, effectors and their interactions [21], collectively referred to here as GPCR signaling pathway proteins. Table 1 lists the key GPCR signaling pathway proteins included in this study. The best diatom sequence match for a human GPCR signaling pathway protein was defined as the sequence that had the lowest E-value below the threshold of E < 10^-5^ over a minimum alignment length of 100 amino acids. The matching genomic regions for each GPCR signaling pathway protein identified in the diatoms were then searched against the respective diatom ESTs using BLASTN (default settings). EST matches were considered significant if they aligned to their corresponding genomic sequence at greater than 98% identity.

To search for the G-protein γ-subunit and RGS in the diatoms, the Gγ-subunit (PF00631) and RGS domain (PF00615) Pfams was searched against the conceptually translated diatom genomes using the HMMER3 sequence analysis software package [48]. Expression of the genomic Gγ-subunit was confirmed as above by aligning the extracted genomic region with the respective diatom EST libraries at greater than 98% identity

### GPCR identification

Known and predicted eukaryotic GPCR protein sequences from all GPCR families were downloaded from the GPCRDB and TBLASTN searched against each translated diatom genome using an E-value threshold of 10^-10^ (Figure 1). These predicted protein sequences were entered into the TMD prediction programs HMMTOP [49] and TMMHMM [50] using default settings. Of those sequences with 7 TMDs, only those that corresponded to full length predicted proteins and gene models and that were predicted to have an extracellular N-terminus (i.e. ligand binding domain) were selected for further analyses. Duplicate sequences were removed. These putative diatom GPCR sequences were further verified for evidence of GPCR motifs and conserved domains using the NCBI Conserved Domain Database (CDD) v2.21 [51] and Pfam v26.0 [52] using an e-value threshold of 10^-5^.

A second approach to identify putative diatom GPCRs involved downloading sequence alignments for the GPCR families from the GPCRDB (classes A, B and C) and then converting these seed alignments to HMM profiles to search against a custom microeukaryote database (Additional file 2: Table S2) and the NCBI RefSeq database using HMMER3 (E-value <10^-5^).

The putative diatom GPCR sequences were also searched against their respective ESTs that were generated under different culture treatments (Table 2). We also searched the putative *T. pseudonana* GPCR sequences against transcriptomic datasets representing silicon repletion and starvation [25,53].

### Phylogeny of putative diatom GPCRs

To phylogenetically classify putative diatom GPCRs, a seed alignment was generated that contained the 7 TMD regions of the 16 putative diatom GPCR sequences identified through the BLAST search of the diatom genomes against the GPCRDB and subsequent motif and N- and C-terminus analyses. The diatom TMD sequences were then converted to a HMM profile using HMMER3 and searched against the custom microeukaryote and the NCBI RefSeq databases (E-value <10^-10^). Only those recruited sequences containing 6–8 TMDs were retained. A subset of the recruited sequences from each of the represented class C subfamilies (GABA_B_, metabotropic glutamate, calcium-sensing, vomeronasal, pheromone, odorant and taste receptors) in addition to predicted and hypothetical proteins were selected based on the lowest E-values per NCBI taxa ID and included in a single alignment using MUSCLE [54]. This alignment was used to generate a phylogenetic tree representing all class C GPCR subfamilies. Phylogenetic and molecular evolutionary analyses were conducted using MEGA v.5 [55]. The best-fit model of protein evolution was predicted for the alignment. A maximum likelihood (ML) tree [56] was inferred using the WAG (+F) model of evolution [57], a discrete gamma distribution and 1,000 bootstrap iterations. To determine the best protein model for ML, we used the model selection function within MEGA, based on a neighbor-joining tree and amino acid substitution model. The model with the lowest Bayesian Information Criterion (BIC) was classified as having the highest likelihood score and was used for inferring the tree. Models tested included JTT, Dayhoff, WAG, cpREV, mtREV24 and rtREV, along with the additional frequency options + F,+G and + I.

## Competing interests

The authors declare that they have no competing interests.

## Authors’ contributions

JAP and EMF conceived the study and developed the approach. JAP, MSP and JCW performed the bioinformatics analyses. RBK, JAP and MSP carried out the phylogenetic analyses and interpretation of results. EVA participated in designing the study. JAP wrote the final manuscript. All authors contributed to the drafting and revision of the manuscript. All authors read and approved the final manuscript.

## Supplementary Material

Additional file 1: Table S1 Best predicted protein hits in diatom genomes to human G protein-coupled receptor (GPCR) signaling pathway proteins. GPCRs, Gγ-subunit and RGS were compared to both human and non-human proteins. Data presented as: e-value|percent identity (except for HMM search)|alignment length|best species match (GPCRs only)|best species match GI (GPCRs only). Click here for file

Additional file 2: Table S2 Microeukaryotes included in the custom database and their respective sources within genome and expressed sequence tag (EST) libraries. Click here for file
